# First identification of NDM-5 associated with OXA-181 in *Escherichia coli* from Egypt

**DOI:** 10.1038/emi.2016.24

**Published:** 2016-04-06

**Authors:** Doaa Gamal, Marta Fernández-Martínez, Inas El-Defrawy, Alain A Ocampo-Sosa, Luis Martínez-Martínez

**Affiliations:** 1 Service of Microbiology, University Hospital Marqués de Valdecilla-IDIVAL, Santander 39008, Spain; 2 Medical Microbiology Department, Theodor Bilharz Research Institute (TBRI), Cairo 12411, Egypt; 3 Department of Molecular Biology, School of Medicine, University of Cantabria, Santander 39008, Spain

**Dear Editor,**

NDM-5, a variant of NDM, was first identified in 2011 in an *Escherichia coli* isolate from the perineum and the throat of a patient in the United Kingdom with a recent history of hospitalization in India.^1^ Three years later, *E. coli* producing the same type of enzyme was isolated from urine and blood specimens of three Algerian patients as the first autochthonous cases of infection.^2^ Just recently a NDM-5-producing *E. coli* isolate was also obtained from the urine of a Spanish patient with pyelonephritis who had no recent history of travelling or hospitalization.^3^ Hereby, we characterize the first case of *E. coli* producing NDM-5, isolated from the ascitic fluid of a 52-year-old female patient with post-hepatitis cirrhosis admitted to Theodor Bilharz Research Institute, a tertiary care hospital in Egypt. The patient had fever, jaundice and massive ascites, and she did not have a travel history. The *E. coli* isolate was identified using Vitek2 system (bioMerieux, Marcy L'Etoile, France) and was resistant to all β-lactams, including carbapenems. The minimum inhibitory concentration (MIC) of a set of antibiotics was determined by the broth microdilution method following the Clinical Laboratory Standards Institute (CLSI) guidelines.^4^ The isolate showed resistance to ceftazidime, cefotaxime, imipenem, meropenem, ertapenem, gentamicin, tobramycin, ciprofloxacin and nalidixic acid, whereas the susceptibility to amikacin, tigecycline and colistin was retained (Table 1). The PCR and sequence analysis for carbapenemases, extended-spectrum *β*-lactamases (ESBLs), plasmid-mediated AmpC cephalosporinase-encoding genes, plasmid-mediated quinolone resistance genes, aminoglycoside-modifying enzymes and methyltransferases^5, 6, 7^ identified the presence of *bla*_NDM-5_*, bla*_CTX-M-15_*, bla*_OXA-181_*, bla*_CMY-2_*, aac(3)-IIa* and *aac(6′)-Ib-cr*. PCR-based plasmid replicon typing detected the presence of two replicons, FIA and FIB.^6^ Multilocus sequence typing (MLST), performed according to the Genotyping of Pathogens and Public Health (Institute Pasteur, Paris, France; http://www.pateur.fr/recherche/genopole/PF8/mlst/Kpneumoniae.html), identified the isolate as ST410, which is different from those in previously reported cases of NDM-5, which were ST648 and ST2659 and was infrequently encountered in North Africa.^8^ Whereas an association between ST410 and NDM-1 has been reported in Norway, the United Kingdom, Switzerland, France and the United States, more recently, it was found in Poland in a patient who had previously received care in Tunisia after a terrorist attack.^8, 9^ To the best of our knowledge, this is the first report of ST410 in Egypt. The genetic environment of *bla*_NDM-5_, assessed by PCR mapping as previously described,^10^ showed 99% similarity to that of the plasmid pHC105 (accession number KM 598665.1), retaining the same gene arrangement (ΔIS*Aba125_ bla*_NDM-5__*ble*_MBL_).^3^

Conjugation experiments using the azide-resistant *E. coli* J53 as the recipient strain at two different temperatures and with plates containing meropenem at 0.5 μg/mL failed. Therefore, plasmid extraction using a QIAGEN Midi Kit (Qiagen, Hilden, Germany) followed by electrotransformation experiments into *E. coli* Top10 was performed.

The resistance phenotype and the gene content of the transformants were assessed and compared with those of the parental cells (Table 1).

Plasmid analysis by an S1 nuclease digestion of the whole genomic DNA followed by pulsed-field gel electrophoresis (S1-PFGE) showed that the isolate had three plasmids of sizes approximately 48.5, 88 and 100 kb. Southern blot hybridization of the S1-PFGE plasmid DNA was performed using a DIG DNA Labeling and Detection Kit (Roche, Mannheim, Germany) with DIG-labeled probes for *bla*_NDM-5_, *bla*_OXA-181_, *bla*_CTX-M-15_, FIA and FIB showing that the *bla*_NDM-5_ and *bla*_CTX-M-15_ were all located on the same plasmid (an ≈100 kb plasmid), in both the parental and the transformed cells, which were also collocated with FIA and FIB, indicating the presence of a multireplicon plasmid, whereas *bla*_OXA-181_ was found on another plasmid of size ≈48.5 kb only in the parental cell.

In Egypt, NDM-1 was first identified in 2013 in one *Klebsiella pneumoniae* isolate, and then more cases were found in Enterobacteriaceae, *Pseudomonas aeruginosa* and *Acinetobacter baumannii*.^11^ By contrast, NDM-2 was previously reported in *A. baumannii* in 2011.^12^ Compared with NDM-1, NDM-5 has two amino-acid substitutions (Val_88_→Leu) and (Met_154_→Leu), which confer enhanced hydrolytic activity against carbapenems.

Additionally, OXA-181, a variant of OXA-48, is associated with other carbapenemase genes, such as *bla*_NDM-1_ and *bla*_VIM-5_.^13^ The co-production of OXA-181 with NDM-5 has been recently reported in *K. pneumoniae*;^14^ however, the emergence of this co-existence in *E. coli* is alarming as it is believed that the worldwide spread of this enzyme is a mirror image to that of NDM-1.^13^

We hereby report the first case of NDM-5 in Egypt, confirming the pervasiveness of the NDM enzymes in North Africa and the urgent need for public health concern towards the evolution and spread of these enzymes.

## Figures and Tables

**Table 1:** MICs of different antibiotics in NDM-5-producing *E. coli* (donor), *E. coli* top10 (recipient), and derived transformants and resistance gene content in parental *E. coli* and the transformant cell

**Isolate**	**MIC of antibiotics**												**Resistance gene content**
	**CAZ**	**CTX**	**IMP**	**MEM**	**ERT**	**AMK**	**GEN**	**TOB**	**CIP**	**NAL**	**TGC**	**CST**	
*E. coli*	>128	>128	16	64	128	4	128	64	>128	>128	0.125	0.125	*bla*_NDM-5_*, bla*_CTXM15_*, bla*_OXA-181_*, bla*_CMY-2_*, FIA, FIB, aac(6′)-Ib-cr, aac(3)-IIa, qnrS*
*E. coli* Top10	0.25	⩽0.06	0.25	⩽0.06	0.002	2	0.5	0.5	⩽0.06	2	0.125	⩽0.06	—
Transformant	>128	>128	4	4	4	4	128	32	⩽0.06	2	0.125	⩽0.06	*bla*_NDM-5_*, bla*_CTX-M-15_*, FIA, FIB, aac(6')-Ib-cr, aac(3)-IIa*

Abbreviations: AMK, amikacin; CAZ, ceftazidime; CIP, ciprofloxacin; CST, colistin; CTX, cefotaxime; ERT, ertapenem; GEN, gentamicin; IPM, imipenem; MEM, meropenem; NAL, nalidixic acid; TGC, tigecycline; TOB, tobramycin.
